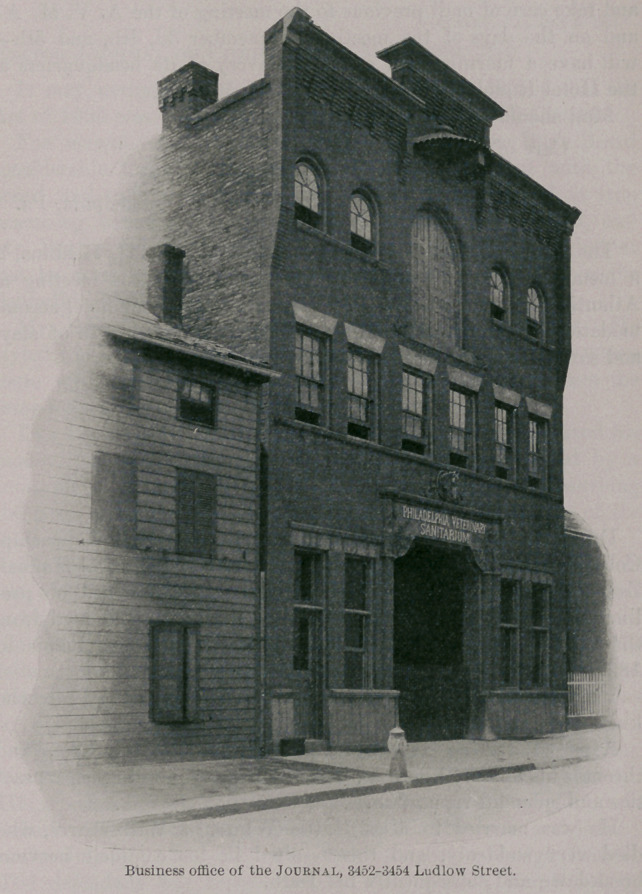# The “Journal’s” Welcome

**Published:** 1901-08

**Authors:** 


### THE "JOURNAL'S" WELCOME.

As the coming annual meeting of the American Veterinary Medical Association will bring an unusual number of veterinarians

Editorial office of the JOURNAL, 1304 Sansom Street.

to and through Philadelphia, on their way to and from Atlantic City, the editors of the JOURNAL take great pleasure in extending a special invitation at this time, and beg to offer whatever courtesy they can give to their visiting colleagues.

The business office of the JOURNAL is at 3452–3454 Ludlow Street, within a few blocks of the Veterinary Department of the University of Pennsylvania.

Business office of the JOURNAL, 3452-3454 Ludlow Street.

A trolley ride to this office by the Market Street cars or the Forty-ninth and Chester Avenue cars on Walnut Street gives a good view of the Schuylkill River and the handsome residential portion of Walnut Street and West Philadelphia. The editorial office, at 1304 Sansom Street, is within two blocks of the central railroad stations of the Pennsylvania and Reading Railroads.

The JOURNAL wishes especially to offer to the visiting veterinarians an address for their mail. It will be prepared to receive and take care of mail previous to the meeting of the A. V. M. A., and on the days of the meeting—September 3d, 4th, and 5th—will have a morning and evening delivery at its headquarters at the Hotel Rudolf at Atlantic City.

Mail should be addressed

Care of JOURNAL C. M. and V. A.,
1304 Sansom Street,
Philadelphia, Pa.

The managing editor of the JOURNAL, Dr. W. H. Hoskins, is a member of the Committee of Arrangements of the meeting at Atlantic City, and will be glad to secure quarters or hotel accommodations upon request, stating number of party, length of stay, and sort of accommodations wanted.

## Figures and Tables

**Figure f1:**
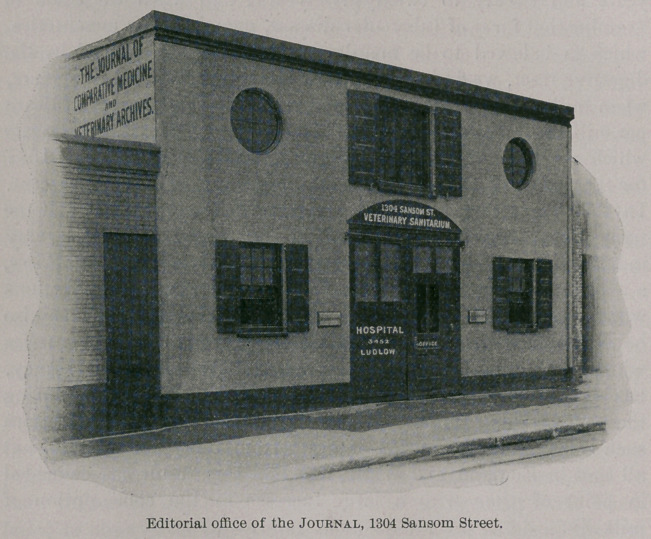


**Figure f2:**